# Structural Evaluation of Steel/CFRP Hybrid Part Using Progressive Damage Model and Cohesive Zone Model

**DOI:** 10.3390/ma18235382

**Published:** 2025-11-28

**Authors:** Jae-Chang Ryu, Min-Gi Kim, Joon-Young Seo, Chan-Joo Lee, Do-Hoon Shin, Dae-Cheol Ko

**Affiliations:** 1 Industrial Liaison Innovation Center, Pusan National University, Busan 46241, Republic of Korea; 2 Department of Nanomechatronics Engineering, Pusan National University, Busan 46241, Republic of Korea; 3 Advanced Mobility Components Group, Korea Institute of Industrial Technology, Daegu 42994, Republic of Korea; 4 Aerostructure Business Department, Korea Air Lines, Busan 46712, Republic of Korea

**Keywords:** hybrid part, impact analysis, progressive damage analysis, cohesive zone model

## Abstract

Carbon-Fiber-Reinforced Plastic (CFRP) is a typical lightweight material used in the aerospace industry. However, the automotive industry has focused on the application of composite materials in vehicle components for weight reduction. In particular, hybrid parts consisting of CFRP reinforcement and a steel outer have been investigated in many studies as a solution to satisfy weight reduction and high strength. In this paper, a steel/CFRP hybrid part was evaluated by impact analysis using several material models, such as the Johnson–Cook model, progressive damage analysis (PDA), and cohesive zone model (CZM). First, the mechanical properties of the steel were determined under different strain rates to assess collision effects. Subsequently, the material properties of the CFRP were evaluated to predict the failure of composite material in the tensile and compressive directions. In addition, the cohesive properties of adhesive film were evaluated under normal and shear modes. Finally, impact analysis using the obtained material properties was conducted to predict the behavior and strength of the steel/CFRP hybrid part under collisions, and the results were compared with the experimental results for verification.

## 1. Introduction

In recent decades, the automotive industry has focused on electronic and hybrid vehicles owing to environmental regulations. However, these vehicles require additional components, such as batteries, which increase their weight. Therefore, the automotive industry has been investigating the application of lightweight materials to components. Carbon Fiber Reinforced Plastics (CFRP), which are used in the aerospace industry, have been investigated owing to their high specific strength [1,2].

Particular attention has been paid to hybrid parts composed of a composite reinforcement and steel outer to reduce weight while maintaining structural strength [3,4]. The strength of hybrid parts depends on several material properties, such as the plastic deformation of steel, failure of the composite, and delamination at the interface of the steel and composite. Kim et al. investigated a progressive damage model of Glass Fiber Reinforced Plastic (GFRP) to predict damage occurrence under various impact conditions [5]. They conducted a finite element (FE) simulation and confirmed the feasibility of a progressive damage model for predicting the crack progress in GFRP plates through verification with experimental results. Lee et al. manufactured two types of reinforcements using a tailor-welded blank and CR420/CFRP and compared them with an FE simulation [6]. A comparison of the results indicated that the reinforcement using CR420/CFRP improved in strength and was lighter in weight. However, delamination at the interface of the hybrid part was not considered. Hu et al. investigated the damage progress of DP590/CFRP hybrid laminate under bending and tensile conditions [7]. A damage model based on the strain for CFRP and a Johnson–Cook model for steel were employed to conduct an FE simulation. The FE results were compared with experimental results for verification. Zhang et al. investigated the impact behavior of steel/CFRP hybrid laminate [8]. An FE simulation was performed to evaluate the effects of various parameters, such as punch velocity, incident angle, and thickness, on the impact resistance. In addition, the performance of a hybrid laminate was evaluated based on the maximum load, damage area, and delamination area. Dlugosch et al. investigated the crashworthiness of a hybrid FRP-steel part under dynamic axial compression [9]. They effectively demonstrated the energy absorption of the hybrid part and limitations of delamination prediction. Although they provided valuable insights into the energy absorption characteristics of hybrid parts, their approach primarily focused on the global structural response rather than on detailed local failure mechanisms, such as adhesive delamination or progressive damage of the composite.

Delamination, which is a critical failure mode at the adhesive interface, can influence the structural performance of hybrid parts under dynamic loads. However, most of the aforementioned studies did not discuss delamination or employ simple geometries. Heshimati et al. investigated the strength of FRP–steel laminates using a cohesive zone model (CZM) [10]. They investigated several effects of parameters, such as type of cohesive law, crack path location, adhesive properties, and FRP properties, on the CZM. Wang et al. conducted an FE simulation and suggested a CZM combined with the user subroutine (UMAT) to describe the interface behavior of the hybrid component [11]. A single-lap test was performed and compared with FE simulation results for verification. Oswaldo et al. performed a double-lap shear test to evaluate the performance of a CFRP–steel specimen [12]. In addition, a non-linear FE model was developed to predict the behavior of specimens with different failure modes. Koord et al. investigated the effects of low temperatures on the interlaminar fracture toughness of steel/CFRP hybrid laminates [13]. They demonstrated the importance and role of thermal residual stresses (TRS) in delamination behavior. However, many studies employed simple geometries or did not consider the damage progression of CFRP despite the significant role of CFRP failure in the reduction in load-carrying capacity.

Although many previous studies have analyzed either the progressive failure of composites or the interfacial delamination of hybrid parts, these approaches have typically been considered independently, leading to limited understanding of the coupled damage behavior under impact loading. Therefore, this study aims to address this gap by combining Progressive Damage Analysis (PDA) and the Cohesive Zone Model (CZM) to predict both the internal damage of CFRP and interfacial delamination simultaneously. This integrated approach provides a more comprehensive understanding of the failure mechanisms and offers a new contribution to the design of hybrid parts under dynamic conditions. First, the material properties of steel, CFRP, and adhesive were determined through various experiments. The steel material was described by the Johnson–Cook model to predict plastic deformation under various strain rates. In addition, the CFRP material was described by PDA to predict failure occurrence under tensile and compressive loads. The behavior of the adhesive was described by CZM under the mixed mode. An impact analysis was performed to predict the resistance to collision and deformed shape of the hybrid part. A steel/CFRP hybrid part was manufactured and employed for impact tests under various impact energies. The impact tests were performed for three cases: CFRP failure only, both CFRP failure and delamination of the adhesive area, and delamination of the adhesive area only. Finally, the load–time curves and shapes between the FE simulations and experiments were compared to verify the results of this study.

## 2. Experiment

### 2.1. Configuration of Steel/CFRP Hybrid Part

**Figure 1:**
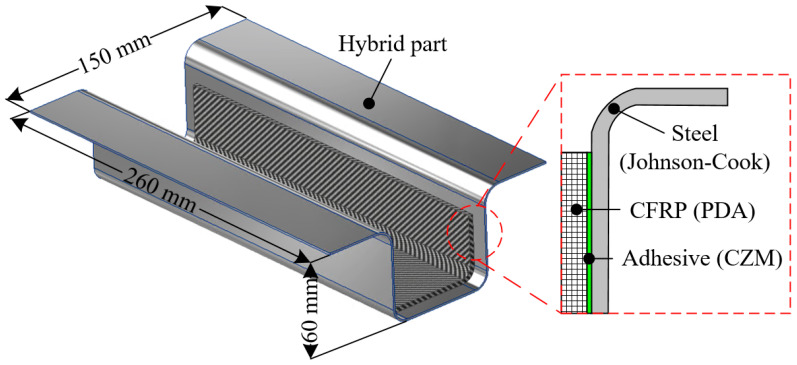
Hybrid part composed of steel, CFRP, and adhesive.

In this work, a steel/CFRP hybrid part representing an automotive B-pillar was manufactured and investigated to predict its deformation behavior under loading conditions. Figure 1 shows the configuration of the steel/CFRP hybrid. The hybrid part was composed of a steel outer, CFRP reinforcement, and adhesive, as shown in the detailed view of Figure 1. The steel outer was made of DP590 (Hyundai Steel, Dangjin, Republic of Korea), and the CFRP reinforcement was fabricated using a plain-weave CFRP prepreg (SK Chemicals, Seongnam, Republic of Korea). As mentioned in the Introduction, the properties of each material should be obtained to describe the deformation behavior of the hybrid part. Therefore, the mechanical properties for the plastic deformation of steel, damage progress of CFRP, and cohesive properties of the adhesive are evaluated in the following sections.

### 2.2. Johnson–Cook Model for DP590 Steel

The Johnson–Cook model has been employed by many researchers to predict the flow behavior of metallic materials according to strain rate and temperature [14,15,16]. The thermal term of the Johnson–Cook model was omitted because both the material tests and impact experiments were conducted under room-temperature conditions. As the study focused on strain-rate-dependent plastic behavior without thermal variations, the simplified form of the Johnson–Cook equation was appropriate for the experimental environment. Therefore, the equation was simplified because there was no variation in temperature. The modified Johnson–Cook equation without thermal effects is as follows:

(1) $$\sigma =\left(A+B\varepsilon^{n}\right)\left(1+Cln\frac{\dot{\varepsilon }}{{\dot{\varepsilon }}_{ref}}\right)$$

where σ and ε are the equivalent stress and equivalent plastic strain, respectively. A, B, n, and C are the yield stress of the material under reference conditions, strain hardening constant, strain hardening coefficient, and strengthening coefficient of the strain rate, respectively [17]. In addition, $\dot{\varepsilon }$ and ${\dot{\varepsilon }}_{ref}$ are the strain rate and reference strain rate, respectively.

**Figure 2:**
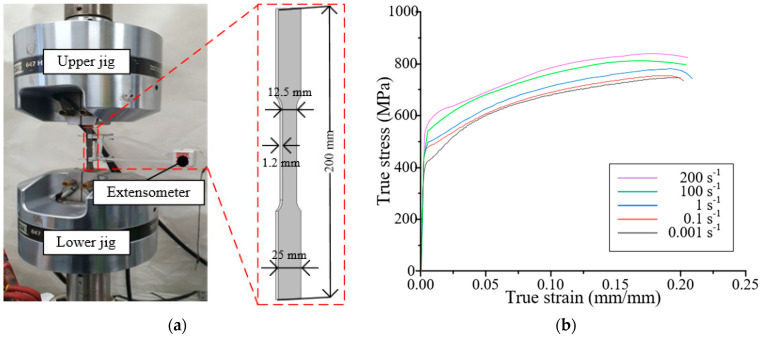
Results of tensile tests according to various strain rates. (**a**) Experimental setup; (**b**) Stress–strain curve.

Uniaxial tensile tests were conducted using the DP590 steel under various strain rates (0.001–200 s^−1^). For each strain rate, three tensile tests were performed, and the obtained stress–strain curve. The specimens were prepared according to the ASTM E8 standard and placed on an MTS universal testing machine (MTS Landmark TM 100 kN, MTS Systems Corporation, Eden Prairie, MN, USA) with a capacity of 100 kN, as shown in Figure 2a. The reference strain rate was taken as 0.001 s^−1^. The stress–strain curves of the tensile tests for several strain rates are shown in Figure 2b. The determined constants A, B, n, and C were 412.9 MPa, 882.8 MPa, 0.5233, and 0.025, respectively. These parameters were directly implemented for the steel outer in the FE model to consider the rate-dependent plastic deformation during impact.

### 2.3. Progressive Damage Model for CFRP

The Hashin criterion was employed to describe the damage mechanism of the CFRP material [18]. The expressions for the onset of damage for fiber tension, fiber compression, matrix tension, and matrix compression are as follows:

(2) $$F_{ft}={\left(\frac{\sigma_{11}}{X_{t}}\right)}^{2}+{\left(\frac{\tau_{12}}{S}\right)}^{2}=1$$

(3) $$F_{fc}={\left(\frac{\sigma_{11}}{X_{c}}\right)}^{2}=1$$

(4) $$F_{mt}={\left(\frac{\sigma_{22}}{Y_{t}}\right)}^{2}+{\left(\frac{\sigma_{12}}{S}\right)}^{2}=1$$

(5) $$F_{mc}={\left(\frac{\sigma_{22}}{2S}\right)}^{2}+\left[{\left(\frac{Y_{c}}{2S}\right)}^{2}-1\right]\frac{\sigma_{22}}{Y_{t}}+{\left(\frac{\tau_{12}}{S}\right)}^{2}$$

Here, $\sigma_{11},\;\sigma_{22}$, and $\tau_{12}$ are the components of the stress tensor along the fiber direction, matrix direction, and shear direction, respectively. $X_{t},\;{\;X}_{c},\;{\;Y}_{t},\;{\;Y}_{c}$, and S are the strengths of fiber tension, fiber compression, matrix tension, matrix compression, and shear direction, respectively.

**Figure 3:**
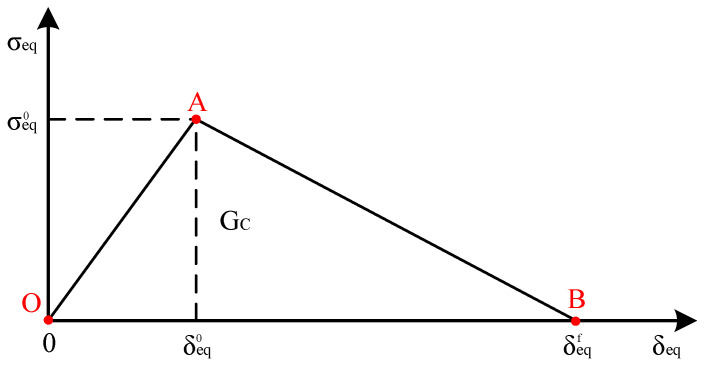
Damage onset and evolution of composite material.

Figure 3 shows the damage onset and evolution for each failure mode of the composite material. Here, $\sigma_{eq}$, $\sigma_{eq}^{0}$, $\delta_{eq}$, $\delta_{eq}^{0}$, $\delta_{eq}^{f}$, and $G_{c}$ are equivalent stress, equivalent stress of damage onset, equivalent displacement, equivalent displacement of damage onset, equivalent displacement at fully damaged, and fracture toughness, respectively. In particular, the element absorbs energy up to point A, indicating damage initiation of the composite material. Beyond point A, damage evolution and stiffness loss occurred until point B, indicating that the element was completely damaged. The triangular area of points O, A, and B corresponds to the fracture toughness of the composite material. To describe the area from point A to point B, the damage variable d was introduced, which was evaluated for the fiber and matrix under tension and compression. The damage variable d is defined as follows:

(6) $$d=\frac{\delta_{eq}^{f}\left(\delta_{eq}-\delta_{eq}^{0}\right)}{\delta_{eq}\left(\delta_{eq}^{f}-\delta_{eq}^{0}\right)}$$

In addition, the equivalent stress and displacement under each mode are summarized in Table 1.

**Figure 4:**
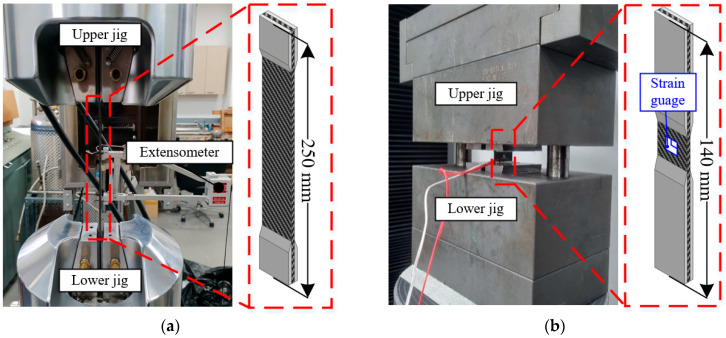
Experimental setup of (**a**) tensile and (**b**) compressive tests.

In the experiments conducted here, various tests were conducted to obtain the material properties of Equations (2)–(5). Tensile tests were conducted using CFRP specimens, which were fabricated with woven thermoset prepregs produced by SK Chemicals according to the ASTM D3039 and ASTM D3518 standards [19,20]. Compressive tests were also conducted to obtain the material properties according to the ASTM D3410 standard [21]. Figure 4 shows the experimental setup used for the tensile and compressive tests.

**Figure 5:**
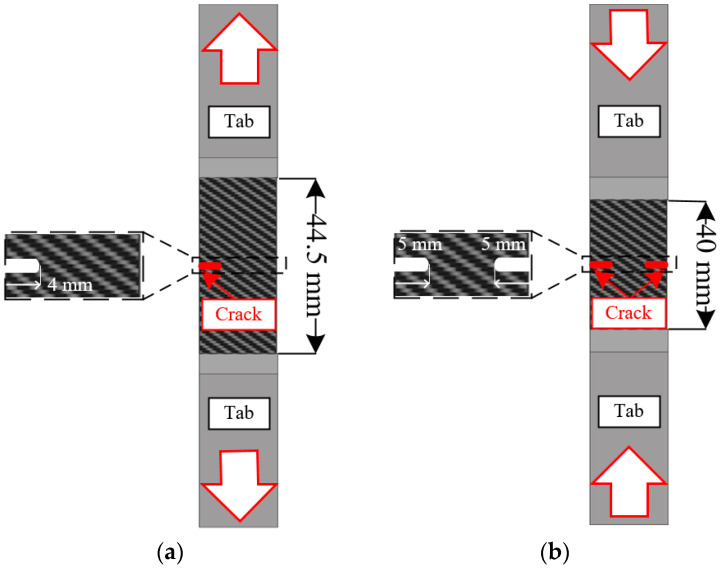
Specimens of (**a**) SENT and (**b**) DENC tests.

Finally, the fracture toughness of the composite material was evaluated considering the loading conditions. Single-edge notched tensile (SENT) and double-edge notched compression (DENC) tests were conducted to obtain fracture toughness of the tensile and compressive modes, respectively. Figure 5 shows each specimen employed for the SENT and DENC tests. All mechanical tests for CFRP were performed at least three times in accordance with ASTM D3039, D3518, and D3410 standards to ensure data reliability. The material properties, which were employed for PDA, are summarized in Table 2.

### 2.4. Cohesive Zone Model for Adhesive

The CZM is widely used to predict delamination of the adhesive area. This method describes the energy release rates based on the traction-separation criterion. The damage criterion is as follows:

(7) $${\left(\frac{<t_{n}>}{t_{n}^{0}}\right)}^{2}+{\left(\frac{t_{s}}{t_{s}^{0}}\right)}^{2}+{\left(\frac{t_{t}}{t_{t}^{0}}\right)}^{2}=1$$

where $t_{n},\;t_{s}$, and $t_{t}\;$ are the normal traction stress and the first and second stresses in the shear direction, respectively. In addition, $t_{n}^{0},\;{\;t}_{s}^{0}$, and $t_{t}^{0}$ are the strengths in the normal direction (mode I) and first and second shear directions (modes II and III), respectively. Beyond damage initiation, damage evolution occurs, and is described by the damage variable *D*. The damage evolution model is expressed as follows:

(8) $$t_{n}=\left\{\begin{array}{cc} \left(1-D\right)\bar{t_{n}}, & \bar{t_{n}}\geq 0\\ \bar{t_{n}}, & \bar{t_{n}}<0 \end{array}\right.\;t_{s}=\left(1-D\right)\bar{t_{s}}\;t_{t}=\left(1-D\right)\bar{t_{t}}$$

Here, $\bar{t_{n}},\;\bar{t_{s}}$, and $\bar{t_{t}}$ are the stress components predicted by the initial traction-separation behavior for the current strain before any damage occurs. To account for the effect of crack closure under compressive loads, the compressive stress does not contribute to the damage for $\bar{t_{n}}<0$. The damage variable *D* progresses from 0 to 1 owing to external loading after damage initiation. When *D* reaches 1, the tractions in both the normal and shear modes become zero. In other words, there is no resistance against deformation of the bonded part. The Benzeggagh–Kenane (BK) law was employed to describe the mixed-mode damage propagation. The fracture toughness $G_{C}$ is expressed as follows:

(9) $$G_{C}=G_{IC}+\left(G_{IIC}-G_{IC}\right){\left(\frac{G_{shear}}{G_{IC}+G_{shear}}\right)}^{\eta }$$

Here, $G_{shear}=G_{IIC}+G_{IIIC}$, and η is the mixed-mode parameter. In addition, $G_{IC},\;G_{IIC}$, and $G_{IIIC}$ are the fracture toughness for modes I, II, and III, respectively.

**Figure 6:**
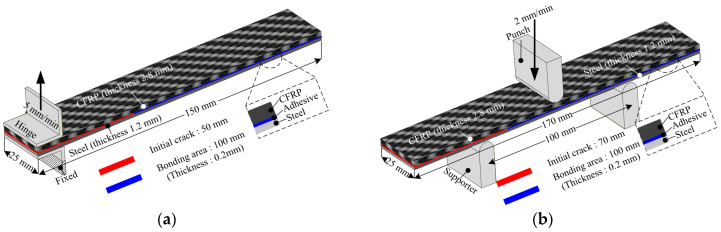
Experimental setup of (**a**) DCB and (**b**) ENF tests for epoxy adhesive film.

**Figure 7:**
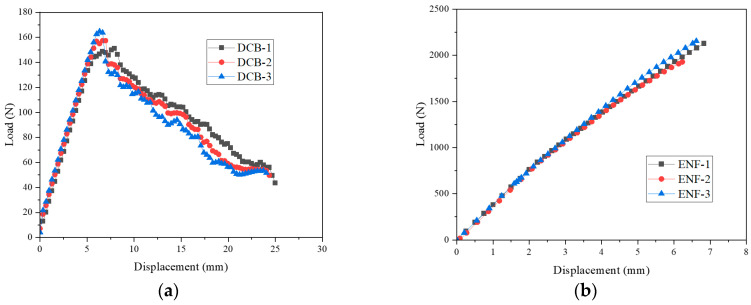
Experimental results of (**a**) DCB and (**b**) ENF tests.

In this paper, two types of adhesive films with different cohesive properties were employed to bond the CFRP reinforcement and steel outer. In general, the strength of a hybrid part depends on the occurrence of delamination during impact conditions. First, the cohesive properties of the epoxy adhesive film were obtained by double cantilever beam (DCB) and end-notched flexure (ENF) tests to consider each loading mode. The specimens were fabricated using a DP590 sheet, CFRP, and epoxy adhesive film. Figure 6 shows a schematic of the experimental setup used for the DCB and ENF tests. The thickness of steel, stacked CFRP, and adhesive were 1.2 mm, 2.8 mm, and 0.2 mm, respectively. The experiments were performed according to the ASTM D5528 and D7905 standards [22,23]. Figure 7 shows the load–displacement curves obtained from the DCB and ENF tests. As a result, the energy release rates of normal and shear directions were obtained as 0.8781 N/mm and 10.4546 N/mm, respectively.

In addition, a different adhesive film was employed to investigate the case mentioned in Section 1, in which only CFRP failure occurs. A rubber adhesive film, which is much stronger than an epoxy adhesive film, was evaluated to obtain the energy release rates. However, excessive bending of the steel adherends and local fracturing of the CFRP were observed during the preliminary DCB and ENF tests. Instead of deriving the cohesive properties experimentally, high energy release rates were adopted as design parameters to realize the no-delamination scenario described in Section 3. In the simulations, the rubber adhesive was assigned as 25.00 N/mm and 30.00 N/mm, respectively. The fracture toughness values of the rubber adhesive were assigned to represent a strong bonding condition because reliable experimental data could not be obtained owing to excessive bending of the steel adherends and local CFRP fracture during preliminary tests. These values were not tuned to achieve specific outcomes but were selected to realize the intended no-delamination scenario.

## 3. Result and Discussion

### 3.1. Impact Analysis of the Hybrid Part

**Figure 8:**
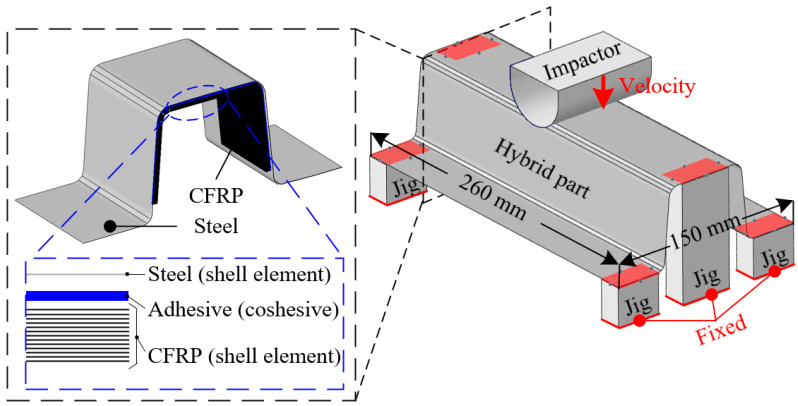
FE model for impact analysis of the hybrid part.

The failures of the adhesive and CFRP reinforcement were evaluated via impact analysis of the steel/CFRP hybrid part using the commercial software ABAQUS 2020. Figure 8 shows the FE model of the hybrid part composed of the steel outer, CFRP reinforcement, and adhesive under impact loading conditions. Here, the four-node shell elements were used for the composite layers of the reinforcement and steel outer. The adhesive film between the steel outer and CFRP layer adopted the eight-node cohesive elements. The mesh size was set to 3 mm for both the steel outer and CFRP reinforcement, and 1.5 mm for the cohesive zone elements of the adhesive layer. A preliminary convergence check confirmed that further refinement did not significantly affect the predicted peak load or delamination area, indicating that the selected mesh density was appropriate for the analysis. The CFRP laminate was modeled with all plies oriented in the 0° direction, as the objective of this study was to evaluate the combined PDA–CZM approach rather than to investigate the influence of layup sequence. The tool set for the impact test comprised an impactor and bottom jigs. The initial velocity was calculated by considering the applied impact energy and weight of the impactor. The hybrid part was placed on fixed bottom jigs. The steel outer, CFRP reinforcement, and adhesive film of the hybrid part were described by the Johnson–Cook model, PDA, and CZM with the material properties obtained in Section 2.

The behavior of the hybrid part under collision is complex because it is composed of a steel outer, CFRP reinforcement, and adhesive. Generally, the strength of the hybrid part during collision depends on the failure of the CFRP and adhesive, as well as the sequence of failure. Therefore, the following three cases should be predicted by FE simulations: Case 1—failure of only the CFRP reinforcement; Case 2—failure of both the CFRP and adhesive; and Case 3—failure of only the adhesive. First, FE simulations were performed to determine the impact energies for the three cases. Based on the simulation results, the impact energies were determined to be 100 and 200 J for Cases 1 and 2, respectively. The hybrid part was composed of the steel outer, CFRP reinforcement, and epoxy adhesive. The impact energy for Case 3 was determined as 300 J. The hybrid part for Case 3 consisted of the steel outer, CFRP reinforcement, and rubber adhesive.

**Figure 9:**
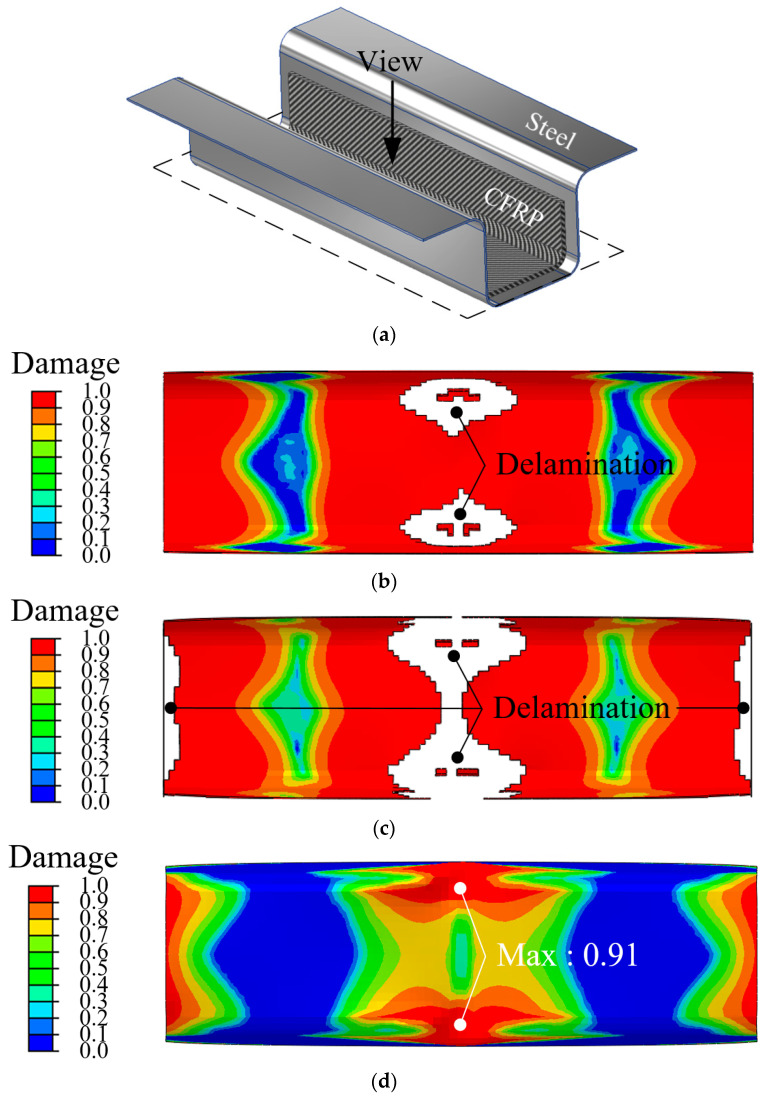

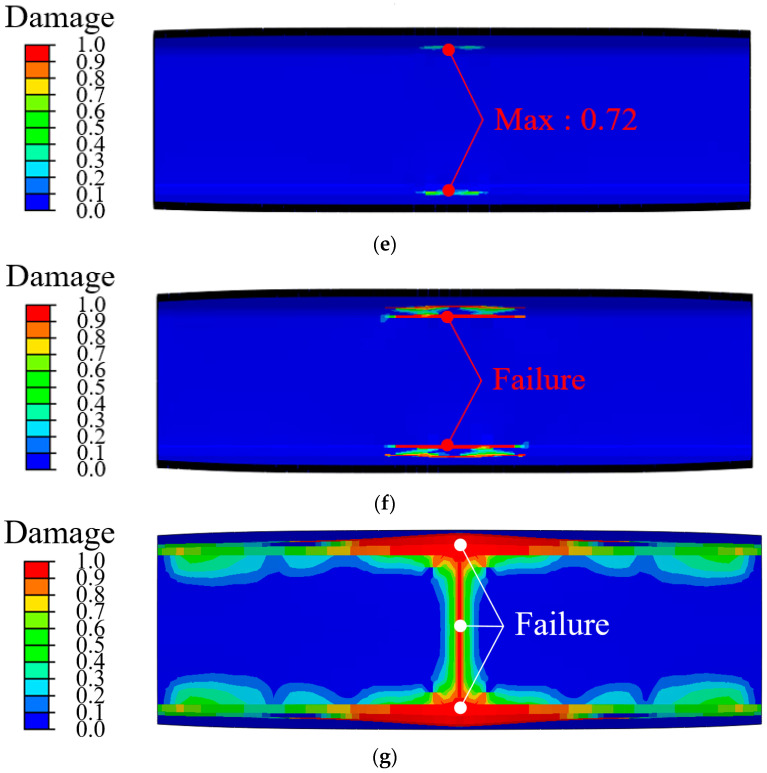
Results of impact analysis. (**a**) View of hybrid part; (**b**) Damage distribution of epoxy adhesive under 100 J impact energy; (**c**) Damage distribution of epoxy adhesive under 200 J impact energy; (**d**) Damage distribution of rubber adhesive under 300 J impact energy; (**e**) Damage distribution of CFRP reinforcement under 100 J impact energy; (**f**) Damage distribution of CFRP reinforcement under 200 J impact energy; (**g**) Damage distribution of CFRP reinforcement under 300 J impact energy.

Figure 9 shows the FE analysis results for the steel/CFRP hybrid part according to various impact energies of 100, 200, and 300 J using different adhesives. Figure 9a shows the direction of view for the results of FE simulations. Delamination of the epoxy adhesive occurred under impact energies of 100 and 200 J, as shown in Figure 9b,c. The damaged region of 200 J was wider compared to that of 100 J. However, the FE simulation using rubber adhesive predicted that the maximum value of damage was 0.91, as shown in Figure 9d. In other words, the steel outer and CFRP reinforcement maintained as bonding state without delamination, despite the higher impact energy of 300 J. This indicates that the superior cohesive properties of the rubber adhesive influenced the resistance capacity to impact load and helped prevent delamination. In Figure 9, the meaning of the damage variable differs depending on the material model. Figure 9b–d show the damage of the cohesive zone, where a damage value of 1 represents complete interfacial separation and results in element deletion. In contrast, Figure 9e–g shows the damage of the CFRP reinforcement obtained from PDA, where a damage value of 1 indicates fully damaged material but the elements remain in the mesh with significantly reduced stiffness. Failure of CFRP reinforcement did not occur in the case of 100 J because the maximum value of damage was 0.72 at the edge, as shown in Figure 9e. However, failure occurred at the edge of the CFRP reinforcement after a bearing impact energy of 200 J, as shown in Figure 9f. Figure 9g illustrates the state of CFRP reinforcement damaged by an impact energy of 300 J. CFRP failure occurred at the center and edge of the reinforcement. In addition, the damaged region was wider than those of 100 and 200 J, as shown in Figure 9e,f. The differences between the epoxy and rubber adhesives were the thicknesses of the reinforcement and delamination. As shown in Figure 9, the performed impact analysis could predict the failure of the CFRP and adhesive, as well as the sequence of these failures under collision conditions.

### 3.2. Impact Test of Hybrid Part

**Figure 10:**
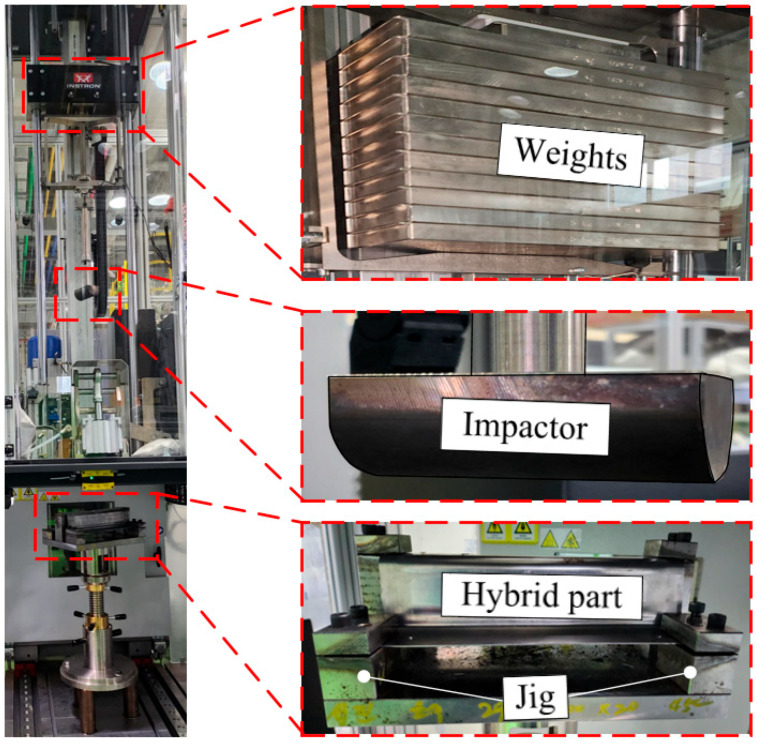
Experimental setup for impact test.

In this study, impact tests were conducted under the same conditions as those used in the FE simulation. Figure 10 shows the experimental setup for the impact test. A drop-weight impact testing machine (Instron CEAST 9450 series) was used. The flange of the hybrid part was constrained by holders and bottom jigs. The distance between the impactor and specimen was 1054 mm. The impact energy was adjusted by adding additional weights to the impactor to consider 100 J, 200 J, and 300 J.

**Figure 11:**
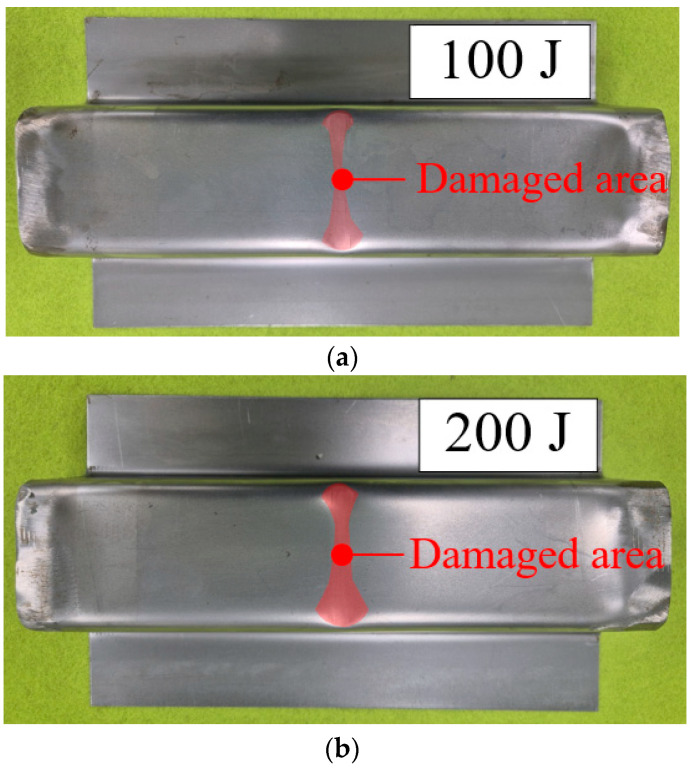

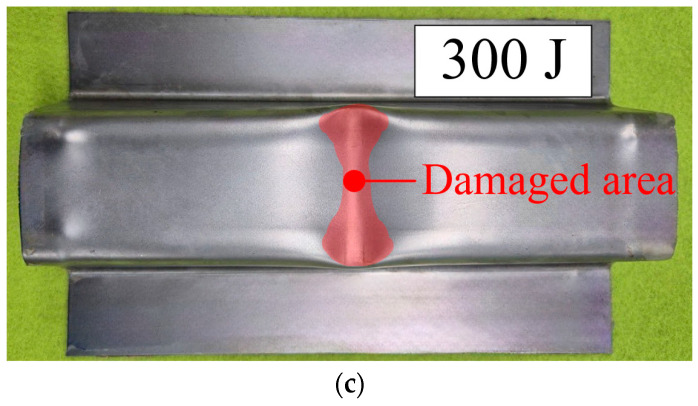
Steel outers after impact tests. (**a**) 100 J; (**b**) 200 J; (**c**) 300 J.

Figure 11 shows the shape of deformed steel outer after the impact tests. In all cases, the steel outers were damaged in center area without any fractures. However, the deformed region of the steel outer became widened as the applied impact energy increased.

**Figure 12:**
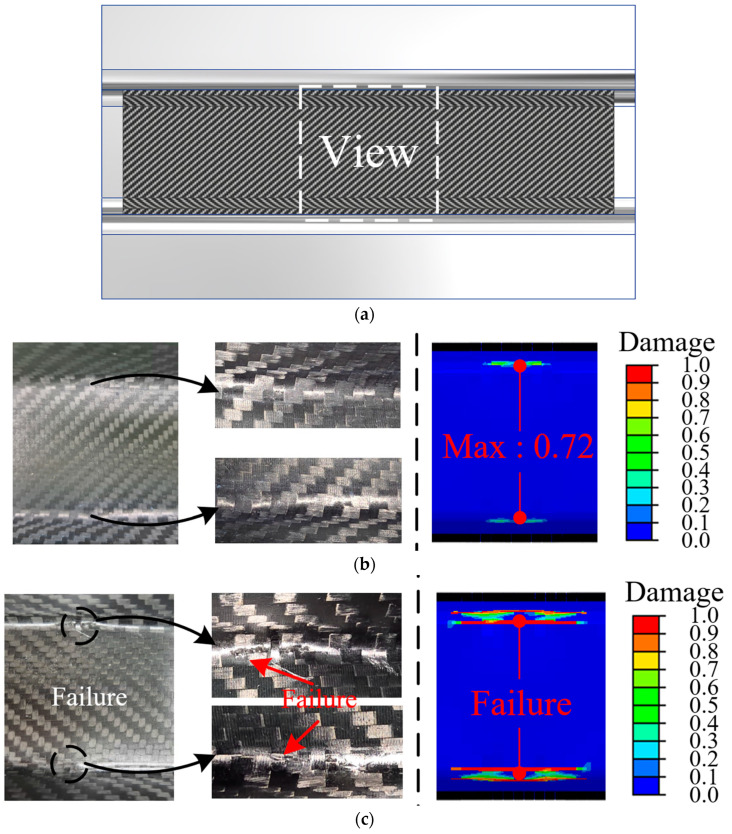

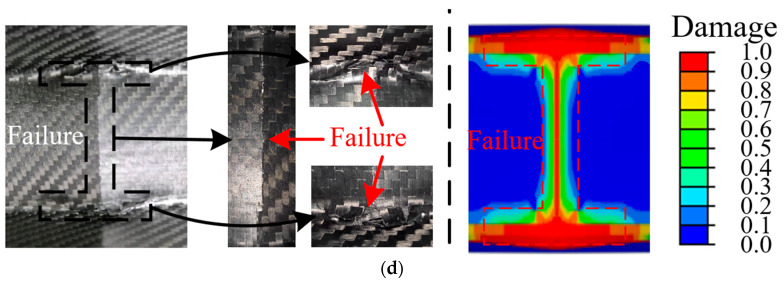
CFRP reinforcements after impact tests. (**a**) View of CFRP reinforcement; (**b**) 100 J (left: experiment, right: FEM); (**c**) 200 J (left: experiment, right: FEM); (**d**) 300 J (left: experiment, right: FEM).

Figure 12 presents the results of the experimental and impact analyses of the CFRP reinforcements at various impact energies. Figure 12a shows the comparison area. Failures did not occur in either the experimental or FE results for the 100 J case, as shown in Figure 12b. Cracks in the CFRP reinforcement were not observed in the experiment for the 100 J case. In addition, the maximum damage value was 0.72 in the FE results of 100 J case, indicating that failure of CFRP did not occur. The CFRP reinforcements began to exhibit localized damage and failure in both the experimental and FE results, as shown in Figure 12c. The consistency between the experimental and FE results indicated that impact analysis using PDA was reasonable for predicting the onset of damage progress of CFRP. In the case of 300 J, the damaged region of the CFRP reinforcements was wider than that of the former in both the experimental and FE results, as shown in Figure 12d. The agreement between the experimental and FE results demonstrates that the FE simulation in this study can help predict the damage progression of CFRP under a high impact energy.

**Figure 13:**
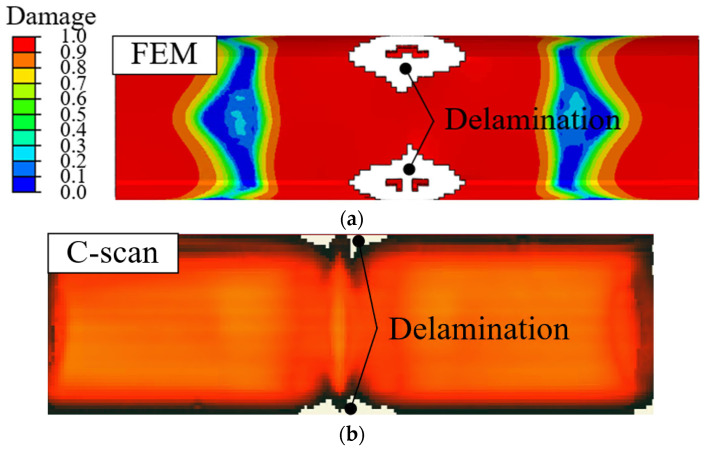

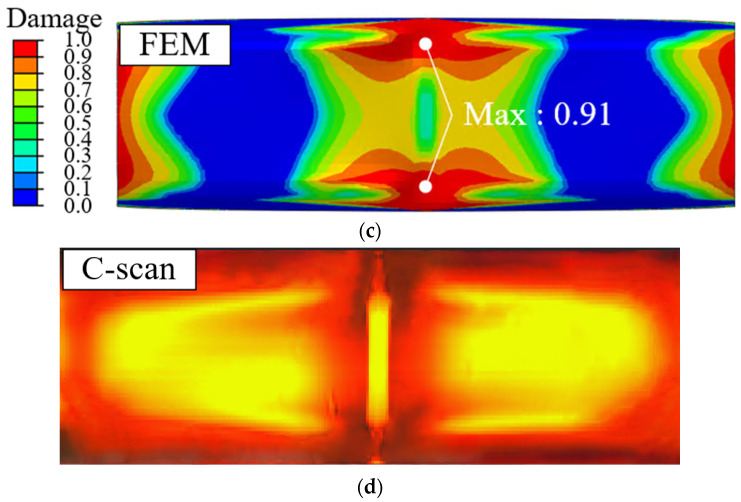
Comparison of c-scan and FEM. (**a**) FE result of 100 J impact analysis; (**b**) c-scan result of 100 J impact test; (**c**) FE result of 300 J impact analysis; (**d**) c-scan result of 300 J impact test.

Figure 13 presents comparative visualizations of the adhesive in the experimental and impact analyses under impact energies of 100 and 300 J. The states of the adhesives were evaluated by c-scan using Immersion Pulse Echo (MATEC, Northborough, MA, USA). The results of the experiments and FE simulations were similar under a low impact energy of 100 J, as shown in Figure 13a and Figure 13b, respectively. The signal loss in the FE simulation and c-scan, which indicated delamination, was positioned at the edges of the hybrid part. The agreement between the experimental and FE results indicated that the impact analysis is useful for predicting delamination under low impact energy. Impact analysis using the rubber adhesive predicted that delamination would not occur despite the high impact energy of 300 J, as shown in Figure 13c. The c-scan result of 300 J was similar to that of FE simulation. The consistency of these results indicated that the prediction of delamination using strong adhesive was reasonable for high-impact energy scenarios.

**Figure 14:**
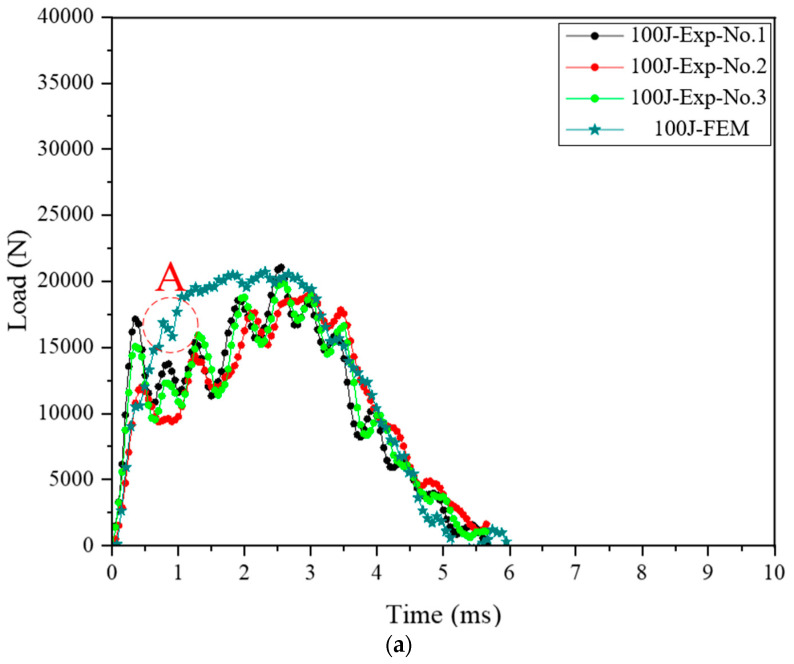

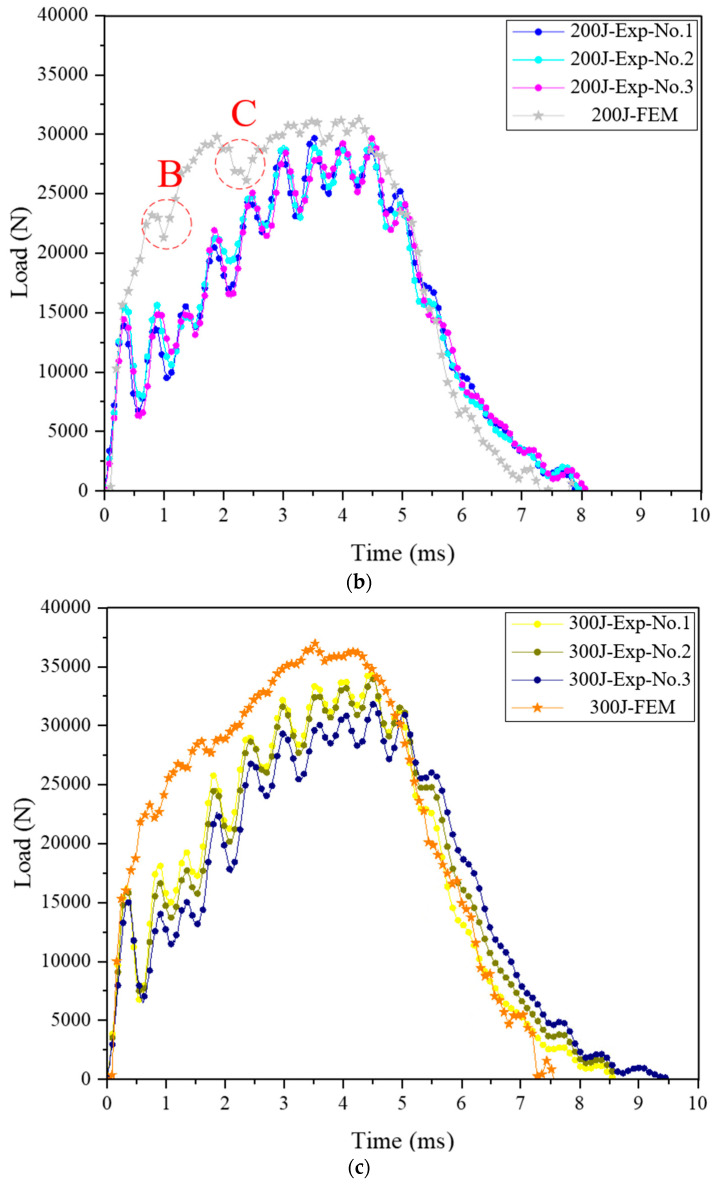
Load–time curves of experiments and FE simulations. (**a**) 100 J; (**b**) 200 J; (**c**) 300 J.

**Figure 15:**
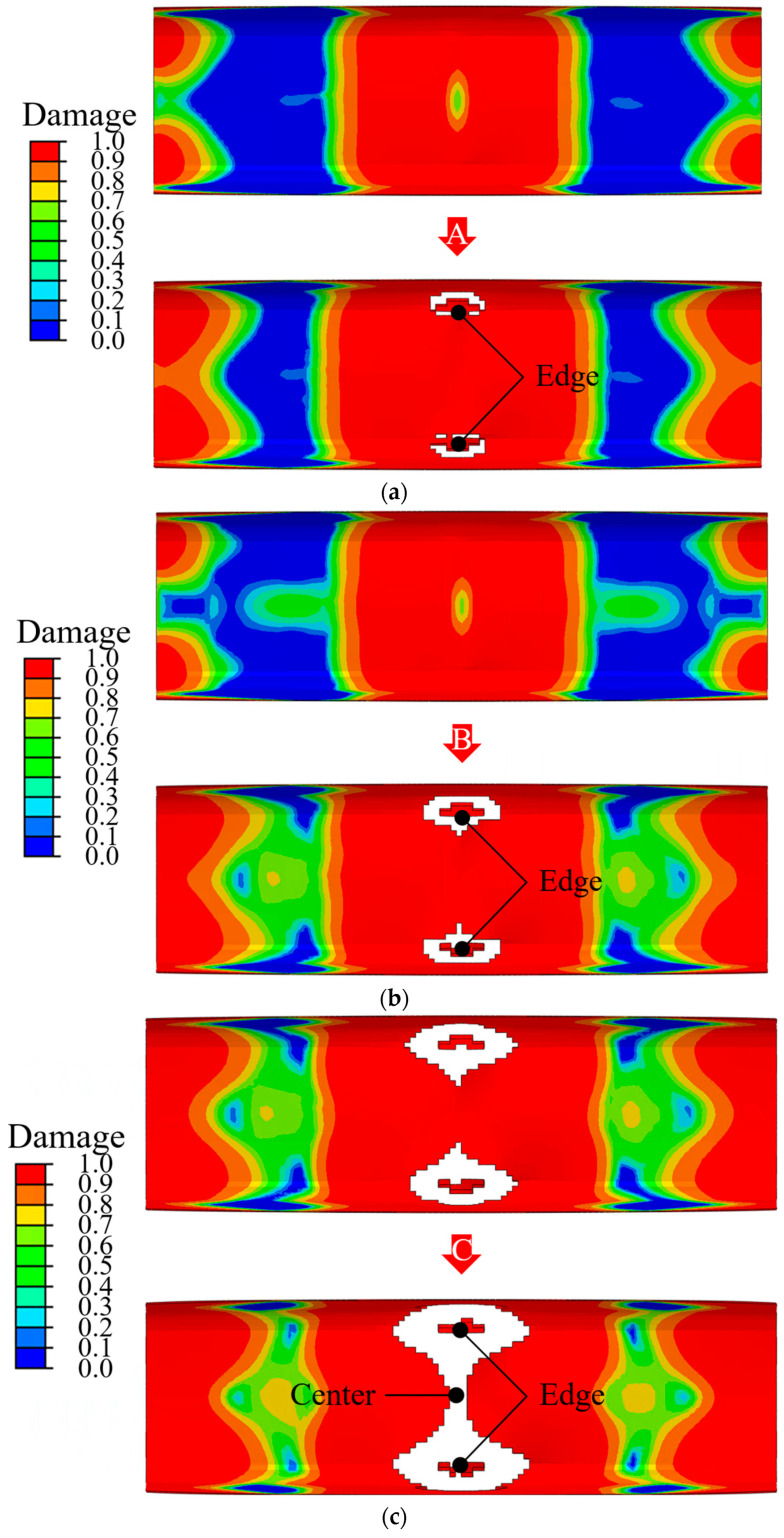
Deformation of cohesive zone at points (**a**) A, (**b**) B and (**c**) C.

Figure 14 presents the load–time curves obtained from the three repeated experiments for each impact energies and FE simulations under impact energies of 100, 200, and 300 J. In addition, Figure 15 illustrates the deformation of the cohesive zone after points A, B and C of Figure 14. A comparison of the experimental and FE results under an impact energy of 100 J is shown in Figure 14a. The experimental and FE simulation curves show a rapid increase in load during the initial impact phase. The load decreased temporarily at point A owing to delamination. Delamination at the edge of the adhesive was initiated and extended beyond point A, as shown in Figure 15a. The peak load values were similar, with a slightly overestimated value predicted by the FE simulation. Most of the experimental and FE simulation curves exhibited a sharp drop after the peak load. The similarity in the load-drop behavior after the peak load confirmed the accuracy of the FE model under low impact energy. Figure 14b illustrates the results of the experiments and FE simulation under an impact energy of 200 J. Most curves increased rapidly during the initial phase of collision. The load curve of the impact analysis decreased twice at points B and C. The first delamination occurred at the edges of the epoxy adhesive, and point B represented this moment, as shown in Figure 15b. Edge-initiated delaminations progressed inward and converged at the center of adhesive film, as shown in Figure 15c. This behavior can be explained by the interaction among the steel outer, adhesive layer, and CFRP reinforcement. Local bending deformation of the steel outer caused an interfacial stress rise in the adhesive layer, which initiated delamination at the edge. Once delamination occurred, a temporary loss of contact between the steel outer and CFRP reinforcement followed, reducing the load-transfer capability at that moment. After contact between the steel outer and CFRP reinforcement was re-established, the transmitted load began to rise again toward the peak value. This process explains the temporary load drops at points B and C and the subsequent recovery of the load curve. The renewed contact also caused a redistribution of load, which promoted further damage progression in the CFRP reinforcement. The peak load values were almost the same, with a slightly higher value predicted by the impact analysis. Most curves at the load drop phase were very similar after reaching the peak load. The agreement of these results verified the FE model using epoxy adhesive under a high impact energy of 200 J. For the 300 J case, the shapes of the curves were similar from the initial phase to peak load value, as shown in Figure 14c. The curve of the FE simulation under an impact energy of 300 J increased without a remarkable drop until the peak load value, unlike in the cases of 100 and 200 J. The absence of delamination was presumed to be the reason for this phenomenon. The curve of the impact analysis in the drop phase was similar to that in the experiments. The agreement between these curves verified that the presented FE simulation using a strong adhesive was acceptable for predicting the behavior of the hybrid part under collision. As a result of comparisons between the experiments and FE simulations, impact analysis was found to be an effective method for predicting the load increase at the initial phase, values of peak load, and locations of the drop phase. In addition, the temporary load drop owing to delamination could be predicted by impact analysis.

In this study, impact analysis using the Johnson–Cook model, PDA, and CZM was conducted to predict the complex behaviors of a steel/CFRP hybrid part. The impact analysis used for this work could improve the methods employed in previous studies. Lee et al. designed a CFRP reinforcement to substitute existing steel parts using structural analysis [24]. However, the employed structural analysis was conducted in the elastic deformation area of the part. In other words, previous studies did not consider the damage progression of the composite material. An FE simulation to predict plastic deformations caused by static or dynamic load should be considered as an important method for designing parts of a vehicle. Kim et al. designed a steel/CFRP hybrid part using structural analysis with sticking conditions at the interface between steel outer and CFRP reinforcement [25]. In other words, they did not consider the occurrence of delamination. Typically, when delamination of the adhesive occurs, the reaction force decreases owing to the detachment of the reinforcement, as shown in Figure 14. However, the impact analysis performed here can predict the plastic deformation, damage progress of CFRP, and delamination. This implies that impact analysis is a useful method for designing hybrid parts under crash situations. This approach provides critical insights for ensuring the structural integrity of hybrid parts. By understanding how different components behave together under various loads, designers can make informed decisions to enhance the durability and safety of the final product.

## 4. Conclusions

This study developed and validated an integrated numerical approach to predict the deformation and failure behavior of steel/CFRP hybrid parts under impact loading. The proposed approach successfully captured internal damage progression within the CFRP as well as interfacial delamination through the combined use of Progressive Damage Analysis (PDA) and the Cohesive Zone Model (CZM).

The impact analyses reproduced the experimentally measured load–time responses with strong quantitative agreement. In particular, the FE simulations accurately predicted the peak load levels observed across the 100–300 J impact conditions and captured the timing and magnitude of temporary load drops associated with delamination. Quantitatively, the peak loads obtained from the FE simulations were compared with the average peak loads measured from the three repeated experiments for each case. The differences between the FE predictions and the experimental averages were 2.92% at 100 J, 5.47% at 200 J, and 8.38% at 300 J, and the FE peak loads were consistently higher than the corresponding experimental values. These results indicate that the proposed approach can reproduce the magnitude and the trend of the structural response across different impact severities. The model also reflected the experimentally observed transition from delamination-driven response at lower energies to continuous load transfer at higher energies, demonstrating its ability to represent different failure behaviors across a range of impact conditions. This capability provides a comprehensive understanding of how adhesive behavior and composite damage interact to define the global structural response under collision.

Overall, the results confirm that the proposed PDA–CZM-based numerical framework offers a reliable and physically consistent tool for assessing the crashworthiness of steel/CFRP hybrid components. Because the methodology can reproduce the failure sequence, peak load evolution, and delamination onset observed in experiments, it can serve as a practical basis for the virtual evaluation and design of lightweight hybrid structures across various impact scenarios and material configurations in automotive and aerospace applications.

## Figures and Tables

**Table 1:** Equivalent stress and displacement.

Loading Mode	Equivalent Stress	Equivalent Displacement
Fiber tension	$\sigma_{eq}^{ft}=\frac{<\sigma_{11}><\varepsilon_{11}>+\tau_{12}\varepsilon_{12}}{\left(\delta_{eq}^{ft}/L_{C}\right)}$	$\delta_{eq}^{ft}=L_{C}\sqrt{{<\varepsilon_{11}>}^{2}+\varepsilon_{12}^{2}}$
Fiber compression	$\sigma_{eq}^{fc}=\frac{<-\sigma_{11}><-\varepsilon_{11}>}{\left(\delta_{eq}^{fc}/L_{C}\right)}$	$\delta_{eq}^{fc}=L_{C}<-\varepsilon_{11}>$
Matrix tension	$\sigma_{eq}^{mt}=\frac{<\sigma_{22}><\varepsilon_{22}>+\tau_{12}\varepsilon_{12}}{\left(\delta_{eq}^{mt}/L_{C}\right)}$	$\delta_{eq}^{mt}=L_{C}\sqrt{{<\varepsilon_{22}>}^{2}+\varepsilon_{12}^{2}}$
Matrix compression	$\sigma_{eq}^{mc}=\frac{<\sigma_{22}><\varepsilon_{22}>+\tau_{12}\varepsilon_{12}}{\left(\delta_{eq}^{mc}/L_{C}\right)}$	$\delta_{eq}^{mc}=L_{C}\sqrt{{<-\varepsilon_{22}>}^{2}+\varepsilon_{12}^{2}}$

Here, $L_{C}$ and < > are the characteristic length of the element and Macauley bracket, respectively.

**Table 2:** Material properties for PDA.

Tensile elastic modulus, *E*_11_ *= E*_22_	61.2 GPa
Compressive elastic modulus, *E_C_*_11_ *= E_C_*_22_	50.4 GPa
Shear modulus, *G*_12_	3.1 GPa
Shear modulus, *G*_23_ *= G*_13_	1.3 GPa
Poisson’s ratio, *ν*_12_	0.13
Tensile strength of fiber and matrix direction, *X_t_ = Y_t_*	605 MPa
Compressive strength of fiber direction, *X_c_ = Y_c_*	495 MPa
In-plane shear strength, *S*	105 MPa
Tensile fracture toughness, $G_{f}^{t}$	15.01 N/mm
Compressive fracture toughness, $G_{f}^{c}$	27.2 N/mm

## Data Availability

The original contributions presented in the study are included in the article, further inquiries can be directed to the corresponding author.
